# On Burkholderiales order microorganisms and cystic fibrosis in Russia

**DOI:** 10.1186/s12864-018-4472-9

**Published:** 2018-02-09

**Authors:** Olga L. Voronina, Marina S. Kunda, Natalia N. Ryzhova, Ekaterina I. Aksenova, Natalia E. Sharapova, Andrey N. Semenov, Elena L. Amelina, Alexandr G. Chuchalin, Alexandr L. Gintsburg

**Affiliations:** 1 N.F. Gamaleya National Research Center of Epidemiology and Microbiology, Moscow, Russia; 2 Research Institute of Pulmonology, Moscow, Russia

**Keywords:** Cystic fibrosis, *Burkholderia cepacia* complex, *Achromobacter spp*., Epidemic strain, Microbiome, Whole genome sequencing, Massively parallel sequencing

## Abstract

**Background:**

Microbes infecting cystic fibrosis patients’ respiratory tract are important in determining patients’ functional status. Representatives of Burkholderiales order are the most dangerous. The goal of our investigation was to reveal the diversity of Burkholderiales, define of their proportion in the microbiome of various parts of respiratory tract and determine the pathogenicity of the main representatives.

**Results:**

In more than 500 cystic fibrosis patients, representing all Federal Regions of Russia, 34.0% were infected by *Burkholderia cepacia* complex *(Bcc)*, 21.0% by *Achromobacter spp*. and 12.0% by *Lautropia mirabilis*. *B. cenocepacia* was the most numerous species among the *Bcc* (93.0%), and *A. ruhlandii* was the most numerous among *Achromobacter spp.* (58.0%). The most abundant genotype in *Bcc* was sequence type (ST) 709, and in *Achromobacter spp.* it was ST36. These STs constitute Russian epidemic strains. Whole genome sequencing of strains *A. ruhlandii* SCCH3:Ach33–1365 ST36 and *B. cenocepacia* GIMC4560:Bcn122 ST709 revealed huge resistomes and many virulence factors, which may explain the difficulties in eradicating these strains. An experience of less dangerous *B. cenocepcia* ST710 elimination was described. Massively parallel sequencing of *16S rDNA* amplicons, including V1-V4 hypervariable regions, was used to definite “healthy” microbiome characteristics. Analysis of maxillary sinus lavage of 7 patients revealed infection with Proteobacteria of the same ST as pathogens from sputum, suggesting that the maxillary sinus is a source of infection in cystic fibrosis patients.

**Conclusions:**

Characterization of the Russian epidemic bacterial strains in the sputum and sinuses of cystic fibrosis patients have better defined the importance of Burkholderiales bacteria. This information may aid in the development of effective approaches for treatment of this disease.

**Electronic supplementary material:**

The online version of this article (10.1186/s12864-018-4472-9) contains supplementary material, which is available to authorized users.

## Background

Microbes infecting the cystic fibrosis (CF) patients’ respiratory tracts adversely affect the patients’ functional status. Among more than 200 bacterial species that may be involved in this disease [1], Burkholderiales order representatives are the most dangerous: well-known representatives are *Burkholderia cepacia* complex (*Bcc*) or emerging *Achromobacter spp.*, *Pandoraea spp*., *Lautropia mirabilis, Variovorax spp*. All of them are multidrug resistant, transmissible and biofilm forming, which makes them difficult to eradicate [2]. Cepacia syndrome was described by Isles [3], who have noted that patients infected with *Pseudomonas cepacia* (the name of *Bcc* in 1970–1980 years) have greater impairment of pulmonary function than those with *P. aeruginosa* [3]. Timely and accurate bacteria identification is key to infection containment in CF. Molecular-genetic methods are more effective for Burkholderiales, which are closely related, difficult to cultivate and complicated in taxonomic affiliation. Thus Lipuma [1] named Burkholderiales *“*a challenge for conventional microbiology”. Since MultiLocus Sequence Typing (MLST) became the routine in Russian, in 2012, examination of CF patients’ samples by this method as in classical, so in express version is underway [4, 5], first for *Bcc*, then for *Achromobacter spp*. [6]. Then revealing new Burkholderiales genera has led to the necessity of developing new in-home schemes [7]. Diversity of species and genotypes was estimated for *Bcc* and *Achromobacter spp*., but these characteristics must be under permanent surveillance because of constantly increasing numbers of CF patients in the monitorable group. Russian epidemic strains *B. cenocepacia* ST709 and *A. ruhlandii* ST36 required the closer attention, so we began investigating strains by whole genome sequencing (WGS). Identification of individual pathogens may still be important for choosing treatment strategies in CF, but the entire microbial community of the respiratory tract can act as pathogens [8], so there is need to examine for diversity. The pioneers in culture-independent techniques for respiratory tract samples, Rogers et al. [9], demonstrated the power of bacterial diversity analysis through use of *16S rDNA* sequencing. Next-generation sequencing of *16S rDNA* then became the gold standard in bacterial diversity analysis [10], and changes in microbial community diversity in various stages of disease were identified [11]. Thus, we have decided to apply this technique in chronically Bcc-infected patients to help appreciate the process of microbial community recovery and to search for additional sources of bacterial infection in CF patients.

## Methods

### Biosamples and bacterial strains

Materials for investigation included sputum and tracheal aspirate from more than 500 patients’ cohorts, representing all Federal Regions of Russia, and from maxillary sinus lavage of 7 adult patients. The patients’ ages ranged from 4 months to 67 years (mean, 18.8 years; median, 19.0 years). The samples were collected from patients attending these CF Centers: Research Institute of Pulmonology; National Scientific and Practical Center of Children’s Health; Russian Children’s Clinical Hospital; and Regional CF Centers.

Approval of the Biomedical Ethics Committee of Research Center was obtained in May 2012 (Proceedings Record N1 d.d. 05.17.2012). Adult subjects or parents of minor subjects provided written informed consent.

Ten patients (P1-P10) were included in the microbiome analysis (Additional file 1: Table S4). The patients’ ages ranged from 23 to 36 years. Patients were subdivided into 3 groups according to the lung disease status, which was controlled by the percentage of predicted forced expiratory volume in 1 s (FEV1,%) [12, 13]: a) CF patients mild lung disease (FEV1% > 70%); b) CF patients with a moderate lung disease (70 < FEV1% > 40); c) CF patients with a severe lung disease (FEV1% < 40).

*B. cepacia* and *B. gladioli* strains of new STs were isolated from the sputum samples at the laboratory of biologically active nanostructure by Dr. R.S. Ovchinnikov.

Strain SCCH3:Ach33–1365, kindly provided by Dr. A.V. Lasareva of the National Scientific and Practical Center of Children’s Health, and strain GIMC4560:Bcn122, kindly provided by the laboratory of molecular epidemiology of our Research Center, were used for the whole genome sequencing (WGS).

### DNA isolation

DNA was isolated according to the protocol of the Maxwell 16 Tissue DNA Purification Kit for Maxwell MDX Instrument (Promega). Preparation of genomic DNA for WGS was performed according to the protocols [14].

### Bacteria genotyping

Bacterial identification and genotyping technology were conducted according to two approaches: 1) detailed classical MLST protocols for *Bcc* and *Achromobacter spp*. completed by *16S rDNA* target and in-home elaborated protocols for other emerging Burkholderiales; and 2) two/three-locus express protocols for prompt screening.

The MLST protocol for *Bcc* allows the differentiation of all *Bcc* species and their genotypes or identification of STs [15]. The MLST protocol for *Achromobacter spp*. is suitable for all *Achromobacter* species representatives [16]. The additive target *nrdA*765 allows the immediate determination of strain species. The protocols, alleles’ sequences and software for data analysis are available on the PubMLST site [17]. For detection of *Pandoraea* spp. we used *gltB* and *recA* targets from the *Bcc* MLST scheme. Gene *gyrB* was suitable for revealing *Variovorax paradoxus*. For *Lautropia mirabilis* we used *gltB* target from *Bcc* MLST scheme, and home-elaborated selective target - *phbB* gene, coding 3-ketoacyl-ACP reductase. The amplification was performed with primers phbB_LauF 5’-GCGCATCGCTTACGTGACTG-3′, phbB_LauR 5’-GGATGGTGGACACGATGCGA-3′ and program: 95 °C—3 min, (95 °C—30 s, 60 °C—40 s, 72 °C—1 min) × 35, 72 °C—5 min. The product size was 608 bp. *16S rDNA* gene fragments were amplified according to Voronina et al. [7].

Express protocol for the samples analysis included three targets: *gltB*, *gyrB* and *16S rDNA* and was performed according to Voronina et al. [5].

Polymerase chain reaction (PCR) products were sequenced according to the protocol of BigDye Terminator 3.1 Cycle Sequencing kit for the Genetic Analyzer 3500 Applied Biosystems. The electrophoretic DNA separation was performed in 50-cm capillaries with POP7 polymer.

New MLST alleles and STs were managed by curators of *Bcc* and *Achromobacter spp.* MLST Databases and submitted with the following ids: 1149–1155, 1189–1267, 1501, 1443, 2029–2030 for *Bcc* and 605–629, 773–774 for *Achromobacter spp*. Sequences of *Pandoraea*, *Variovorax* and *Lautropia* genes were deposited in GenBank under the accession numbers: KM410934, KM410935; KJ848780; KT345191 - KT345194. *Achromobacter spp. gltB* gene sequences from express protocols had accession numbers KC817498-KC817502, KF290958, KF290959, KF297891, KF963246- KF963250, KJ364657, KJ439616, KJ941209, KM262752, KM262753, KP027420, KP765437- KP765444, KP940471.

### Lung microbiome analysis

Lung microbiome was analyzed by massively parallel sequencing of *16S rDNA* amplicons based on the MiSeq Illumina platform. *16S rDNA* amplicons, which included V1-V4 hypervariable regions (753 bp), were used for the preparation of paired-end libraries according to the Nextera XT DNA Library Prep Kit protocol, then sequenced by the MiSeq Reagent Kit v3 (600 cycles) according to the supplied protocol. The Microbial Genomics Module of CLC Genomic Workbench v.8.5.1 was used with default settings to perform Operational Taxonomic Unit clustering. Greengenes database v13_5 [18] was used as reference with 97% threshold.

### Whole genome sequencing

WGS with 454 Roche technology was performed for Russian epidemic strains *A. ruhlandii* SCCH3:Ach33–1365 ST36 (AruhST36) and *B. cenocepacia* GIMC4560:Bcn122 ST709 (BcenST709). Shotgun and paired-end libraries were prepared. Paired-end libraries were built according to the 3 kb protocol. The sequencing procedure was performed with a GS Junior Titanium Sequencing Kit, and a GS Junior+ Series XL+ Kit, according to the manufacturer’s guidelines. Genome assembly was performed with 454 Sequencing System Software v.2.7 and v.3.0 (Roche).

### Reference genomes

For assembling genomes, the following references presented in the NCBI Genome database were used: *A. xylosoxidans* strain MN001 (CP012046.1) and *A. denitrificans* strain USDA-ARS-USMARC-56712 (CP013923.1) as the main references; *A. xylosoxidans* C54 (GL636060.1); *A. xylosoxidans* A8 (HE798385.1) and *A. xylosoxidans* strain FDAARGOS_150 (CP014028) as the additional references. The main reference for three chromosomes of BcenST709 was *B. cenocepacia* J2315 (NC_011000.1- NC_011002.1). The additional references for genome assembling were *B*. *cenocepacia* strain 895 (CP015036.1 - NZ_CP015038.1); *B. cenocepacia* strain MSMB384WGS (NZ_CP013450.1 - CP013452.1); *B. cenocepacia* strain ST32 (NZ_CP011917.1 - CP011919.1); *B. cenocepacia* PC184 cont1.51 (AAKX01000051.1); *B. ubonensis* strain MSMB2109WGS (NZ_LPHO01000048.1); *B. cepacia* strain DDS 7H-2 chromosome 3 (CP007785.1).

### Bioinformatic analyses

The software Rapid Annotations Subsystems Technology and SEED [19, 20] were used for genome annotation. Complementary analysis was made with help of conserved domains search services: KEGG [21], KEGG OC [22], COGs [23]; protein subcellular localization prediction software: TMHMM Server v.2.0 [24], SignalP 4.1 Server [25], PSORTb version 3.0.2 [26], InterPro server [27, 28]. Prophage sequences were revealed with help of PHAST (PHAge Search Tool) [29, 30]. WGS data are available in GenBank: Accession Numbers are CP017433 (*A. ruhlandii* SCCH3:Ach33–1365) and CP020599 - CP020601 (*B. cenocepacia* GIMC4560:Bcn122 chr1–3).

### Type III secretion system and effectors prediction

Sequence of type III secretion system (T3SS) gene cluster (Accession Number AY028431) was used for searching the analogs in the BcenST709 genome. Sequences of the proteins annotated in T3SS were the references for identifying T3SS components in the AruhST36 proteome. EffectiveDB server was used for prediction of proteins secreting by T3SS (effectors) [31, 32]. Coiled-coil domains of effectors were identified with COILS server [33, 34]. The potential phosphorylation sites of effectors were predicted by NetPhos 3.1 Server [35, 36].

## Results

### Diversity of the Burkholderiales representatives in Russian CF patients’ respiratory tracts

Burkholderiales species are relevant for Russian CF patients. In an analyzed cohort 34.0% of patients were infected by *Bcc*, 21.0% by *Achromobacter spp*., and 12.0% by *Lautropia mirabilis*. *Pandoraea pnomenusa* was seldom found [37]. Two patients had this bacterium in the respiratory tract, one together with *B. mulivorans* and *Lautropia mirabilis*. *Variovorax paradoxus* was detected in the samples of three patients who have congenital lung malformation and in one nurse from the pulmonary department.

**Fig. 1:**
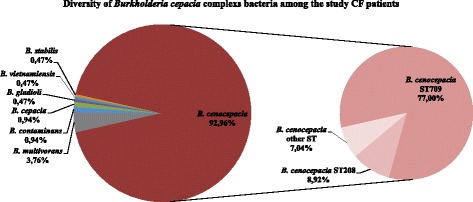
Diversity of *Burkholderia cepacia* complex bacteria among the study CF patients

*Bcc* was represented by 6 species (Fig. 1), the most abundant being *B. cenocepacia* which constituted 93.0% of all *Bcc* cases. Among 19 identified *Bcc* genotypes (Table 1) ST709 was constituted 77.0%. Despite the high *B. cenocepacia* prevalence had not changed since the beginning of the control period, in 2012 [4], the prevalence of ST709 decreased somewhat [7], because the proportion of ST208 increased to 9.0%. The diversity of *Bcc* species and ST increased in 2016–2017 years, when *B. gladioli, B. cepacia* and *B. stabilis* strains were isolated. Interestingly, two *B. cepacia* strains (ST438 and ST1083) were found in the sputum of one patient: the newly registered ST1083 was a double-locus variant of ST438 (*gyrB* and *lepA* genes).

**Table 1:** Genotypes of *Bcc* and *Achromobacter spp*. infecting Russian CF patients

*Bcc*	Sequence type
*B. cenocepacia*	709, 208, 728, 708, 241, 862, 710, 204, 878
*B. multivorans*	711, 712, 835, 195, 659, 783
*B. contaminans*	102
*B. vietnamiensis*	729
*B. gladioli*	903
*B. cepacia*	438, 1083
*B. stabilis*	653
*Achromobacter spp*	ST
*A. ruhlandii*	36, 261, 262, 263, 265
*A. xylosoxidans*	8, 20, 182, 211, 251, 252, 253, 254, 255, 256, 257, 259, 260, 264, 266, 267, 346
*A. dolens*	54
*A. marplatensis*	258, 268
*A. pulmonis*	269
*A. insuavis*	218, 345

**Fig. 2:**
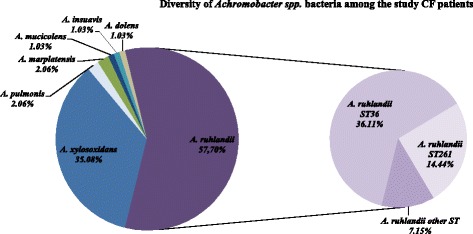
Diversity of *Achromobacter spp.* bacteria among the study CF patients

Among *Achromobacter* species *A. ruhlandii* was the most abundant (58.0%) (Fig. 2). *A. ruhlandii* ST36 was the most abundant *Achromobacter* (35.7%), which was more abundant than all *A. xylosoxidans* genotypes (34.7%) (Table 1). The next most abundant *A. ruhlandii* genotype was ST261 (14.0%).

Retrospective analysis of CF patients revealed the replacement of ST261 by ST36 and nosocomial outbreak of *A. ruhlandii* ST36 infection in the late 1990s in Russian Children’s Clinical Hospital [6]. The bacterium continues circulating among CF patients, among which the youngest infected patient is 3 years old.

*B. cepacia* ST709 and *A. ruhlandii* ST36 are transmissible, are most abundant among Burkholderiales strains, decrease the respiratory function of patients, and have spread into all Russian Federal Regions, except the Far Eastern Region. Thus, these bacteria have been designated Russian epidemic strains.

*B. cepacia* ST709 is the most dangerous: 75% of patients, who have died since beginning of the control, in 2012, were infected by ST709.

We have asked, how often CF patients are co-infected with multiple Burkholderiales representatives. The answer seems to be, seldom: only 1.7% of patients from our cohort were co-infected. The following combinations were identified in patients of various ages: *B. gladioli* ST903 and *A. ruhlandii* ST36 (patient 32 years old); *B. cenocepacia* ST709 and *A. ruhlandii* ST36 (29 years); *A. xylosoxidans* and *L. mirabilis* (19 years); *B. cenocepacia* ST709 and *L. mirabilis* (19 years); *B. multivorans* ST783, *P. pnomenusa* and *L. mirabilis* (19 years); *B. cenocepacia* ST709 and *A. ruhlandii* ST261 (17 years); *B. cenocepacia* ST710 and *A. xylosoxidans* ST182 (4 years). These co-infections have not correlated with respiratory tract function or nutritional status of the patients. It should be noted that in the first example, *B. gladioli* ST903 and *A. ruhlandii* ST36 changed *B. multivorans* ST711 after a long infection period.

### Comparison of two epidemic strains genomes.

To try to understand the reason for the pathogenicity of the Russian epidemic strain *B. cenocepacia* ST709 and *A. ruhlandii* ST36 we performed WGS on them. The features of the epidemic strains’ genomes are presented in Table 2. The BcenST709 genome is bigger and more complicated; it consists of three chromosomes and contains nearly twice the amount of insertion sequence (IS) elements and prophages, two of which are intact (Table 2, Additional file 2: Table S1, Additional file 3: Table S2). In contrast, the clustered regularly interspaced short palindromic repeats (CRISPR), which are elements of bacterial immune systems, were predicted only in the AruhST36 genome. As a result, AruhST36 may be better protected from foreign genetic elements.

**Table 2:** Genome size and number of mobile genome elements of *A. ruhlandii* SCCH3:Ach33–1365 and *B. cenocepacia* GIMC4560:Bcn122

Genome characteristics	*A. ruhlandii* SCCH3:Ach33–1365	*B. cenocepacia* GIMC4560:Bcn122 chr1–3
Accession/ Genome size	CP017433 (6,4 Mb)	CP020599 Bcn122_chr1 (3.5 Mb);
		CP020600 Bcn122_chr2 (3.5 Mb);
		CP020601 Bcn122_chr3 (1.0 Mb)
IS elements	19	37
Prophages	4	7
*intact*	0	2 (26.3Kb; 33Kb)
*incomplete*	3 (18.7Kb; 8.5Kb; 20.1Kb)	5 (21.7Kb; 10.5Kb; 8Kb; 11.6Kb; 19Kb)
*questionable*	1 (32.4Kb)	0
Possible CRISPR	7	0

*IS* insertion sequence, *CRISPR* clustered regularly interspaced short palindromic repeats

Multiple natural antibiotic resistance is a characteristic of Burkholderiales bacteria, which originate from the soil [1]. Huge resistomes of BcenST709 and AruhST36 include beta-lactamases in numbers approximately proportional to the genome size. As illustrated in Table 3, most BcenST709 beta-lactamases are on chromosome 2. Four classes of beta-lactamases were present in the proteomes of epidemic strains, and most of the proteins were from class C, followed by B, A and D, with one protein. An important characteristic of these proteins is resistance to beta-lactam inhibitors.

**Table 3:** Virulence factors and antimicrobial resistance of *A. ruhlandii* SCCH3:Ach33–1365 and *B. cenocepacia* GIMC4560:Bcn122

The component	*A. ruhlandii* SCCH3:Ach33–1365	*B. cenocepacia* GIMC4560:Bcn122 chr1	*B. cenocepacia* GIMC4560:Bcn122 chr2	*B. cenocepacia* GIMC4560:Bcn122 chr3	*B. cenocepacia* GIMC4560:Bcn122 chr1–3
The number					
Antimicrobial agents resistance					
beta-lactamases	10	4	8	1	13
class A	2	0	2	0	2
class B	3	3	1	0	4
class C	4	1	4	1	6
class D	1	0	1	0	1
aminoglycoside phosphotransferase	0	3	0	0	3
aminoglycoside acetyltransferase	2	0	0	0	0
macrolide glycosyltransferase	0	1	0	0	1
number of RND pumps	13	8	9	5	22
number of complete RND pumps	10	5	5	3	13
number of incomplete RND pumps	3	3	4	2	9
acriflavine/ heavy metal resistance RND pumps	5	3	2	1	6
drug resistance RND pumps	3	1	1	2	4
RND pumps with unknown function	2	1	2	0	3
Mobility, adhesion, invasion					
Flp pili (type VIb) proteins	18	14	0	0	14
IVа type pili proteins	0	4	0	0	4
flagella proteins	57	52	0	0	52
capsule proteins	10	14	0	0	14
Type III secretion system					
type III secretion system injectisome complex proteins	32	0	23	0	23
search for secreted type-III proteins (EffectiveT3)	570	314	321	98	733
sorted by Psortb and TMHMM	224	135	157	52	344
with coiled-coil domain	81	47	30	11	88
with MxxY motif	9	6	5	2	13

*RND* Resistance-Nodulation-Division, *Flp* fimbrial low-molecular weight protein, *Psortb* the server for prediction of the protein subcellular localization [26], *TMMH* the server for prediction of transmembrane helices in proteins [24]

Beta-lactamases of class C are more active on cephalosporins than benzylpenicillin, are usually resistant to inhibition by clavulanic acid, and are active on cephamycins, such as cefoxitin [38]. Class B beta-lactamases (metallo-beta-lactamases) determine resistance to almost all beta-lactam antibiotics, including carbapenems [39]. Class A beta-lactamases are represented by two types: PER (Pseudomonas extended resistance) and PenA (penicillin-binding protein 2). The PER type encodes high resistance to cephalosporins (ceftazidime and gentamicin) and intermediate resistance to aminoglycosides (amikacin) [40]. The PenA type, first revealed in *Burkholderia mallei,* determines resistance to penicillins and ceftazidime [41].

Class D beta-lactamases are oxacillinases, but they can hydrolyze both benzyl penicillin and its derivatives (oxacillin, cloxacillin, amino- and carboxypenicillin) [42]. Oxacillinases are genera taxonomic markers in class Proteobacteria [43] and species-specific marker for *Achromobacter spp.* [44]. Allele OXA-258 is typical for *A. ruhlandii*, OXA-114 for *A. xylosoxidans* and *A. marplatensis*, and OXA-364 for *A. dolens* [6].

Aminoglycoside phosphotransferase and macrolide glycosyltransferase amplify the resistome of BcenST709, but only aminoglycoside acetyltransferases complete the resistome of AruhST36.

Active transport is one of the most important mechanisms that determine resistance of microbial cell to antimicrobial agents. We have found that almost 50% of AruhST36 proteins participate in transport of various substances (29.3% of proteome are inner membrane proteins, 21.3% have transmembrane domains and 2.4% are outer membrane proteins). Resistance-Nodulation-Division (RND) family transporters are widespread among gram-negative bacteria. RND are tripartite, which allows them to export drugs directly into the external medium, rather than into the periplasm [45]. The number of RND transporters in AruhST36 and BcenST709 are also proportionate to the genome size. The genes of RND transporters are organized in the operons in both genomes. For all operons, promoter regions have been characterized. But as demonstrated by analysis of the operons, not all RND pumps have complete number of components, so their effective function is questionable. For example, two AruhST36 RND operons (AruCF_0558−AruCF_0559; AruCF_1425−AruCF_1427) encode two components, whereas AruCF_5254−AruCF_5259 operon encodes only one component of the RND pumps. As illustrated in Table 3, nearly half of BcenST709 RND operons are incomplete, so two epidemic strains have equal number of RND pumps with complete function.

A second problem in defining RND transporter characteristics is estimating the numbers of transmembrane domains in the internal membrane components. According to Putman et al. [46], internal membrane components of RND pumps have 12 transmembrane domains, but only 7 RND pumps of AruhST36 meet this requirement: three internal membrane proteins had 11 and 10 transmembrane domains. However, it should be noted that fewer transmembrane domains were present in RND pumps components of *Burkholderia cenocepacia* J2315 also [47]. Thus, proteins with fewer transmembrane domains can be functional.

RND transporters provide resistance to a broad spectrum of substances. For example, three RND transporter operons of AruhST36 encode resistance to cobalt, zinc and cadmium, and two operons encode resistance to the antiseptic acriflavin. RND operon AxyABM provides resistance to cephalosporins (except cefepime), fluoroquinolones and chloramphenicol [48]. This type of RND pump was first described in *A. xylosoxidans*. RND efflux pump CmeABC is common among gram-negative bacteria, determines ciprofloxacine resistance, and acts synergistically with *tet(O*) gene to promote resistance to tetracycline [49]. The other RND-type operon, AxyXY-OprZ, can extrude cefepime, carbapenems, some fluoroquinolones, tetracyclines, and erythromycin from the epidemic strains’ cells. Also AxyXY-OprZ plays a role in the innate resistance to aztreonam [50].

The other way that microbial cells can attain antimicrobial resistance is through modification of lipopolysaccharides. In the case of polymyxin resistance formation, this modification occurs by the addition of 4-amino-4-deoxy-L-arabinose to lipid A, a process that is determined by the arnBCADTEF operon, as described in *P. aeruginosa* [51]. Search in AruhST36 genome for genes with the same function as in arnBCADTEF operon revealed 7 open reading frames (ORF) putatively participating in polymyxin resistance.

In description of bacterial resistance, genes encoding resistance to abiotic substances also must be considered. In the AruhST36 genome, three relevant genes are annotated: for albicidin resistance protein, pyrabactin resistance family protein and for ethidium bromide-methyl viologen resistance protein EmrE. These genes are evidence for soil and plant root systems as an early ecological niche of epidemic strains: albicidin is synthesized by the plant pathogenic bacterium *Xanthomonas albilineans* [52]; and the synthetic sulfonamide pyrabactin mimics abscisic acid, a naturally produced stress hormone in plants that inhibits cell growth.

Numerous capsular proteins, the part of virulence potential responsible for adhesion, are present in both genomes (Table 3).

The next participants in the adaptation and pathogenic function of epidemic strains are pili and flagella. However, flagella cannot be considered without type III secretion system (T3SS) injectisome because of their common origin [53]. The AruhST36 and BcenST709 genomes encode various pili: BcenST709 contains IVа and VIb type pili, and AruhST36 contains only VIb type pili.

The number of flagellar proteins is similar in the proteomes of epidemic strains (Table 3), but the number of chemotaxis proteins is different; only one methyl-accepting chemotaxis protein was revealed in BcenST709, but six chemotaxis proteins were detected in AruhST36.

The T3SSs of these strains are more different. First, the size of gene cluster is bigger in BcenST709 and has a unique sequence, but one gene (*virB1*) of AruhST36 T3SS is located separately. The number of ORF, coding by T3SS gene cluster, is bigger in the AruhST36 genome. The similarity of T3SS injectisome proteins of two epidemic strains is only 28–57%. The analysis based on the traditional ATPase of flagellum sequences suggested the membership of different T3SS families for AruhST36 and BcenST709 T3SSs: the first one belongs to the EscN/YscN/HrcN family, and the second one to the Hrc-Hrp2 family of T3SS. Troisfontaines et al. [53] suggested that the presence of the T3SS Ysc family is an indicator of an extracellular pathogen.

Our searching for effectors of T3SS included four steps (Table 3): predicting secreted proteins with the help of EffectiveDB server; correction of data based PSORTb information, choosing only coiled-coil domain containing proteins, and data-sorting according to MxxY motif in protein sequence (Additional file 3: Table S2). For example, two of AruhST36 proteins (AruCF_0917 and AruCF_5495), selected by this method, had 3 copies of MxxY motif and were involved in heme metabolism. All sorted proteins were candidate effectors that can disrupt key cellular processes of host innate immunity [54, 55].

Thus Russian epidemic strains have huge resistomes and adaptive potential to resist their eradication, and the question of whether resources are available to overcome other genotypes of Burkholderiales must be asked. However, the physicians of Russian Adult CF Center have experience in eradication of *B. cenocepacia* ST710. Lung microbiome analysis became the basis of evidence-based medicine since 2010s for confirmation of therapy efficacy in case of tuberculosis, asthma and other bronchopulmonary diseases. We used microbiome analysis for examination of Burkholderia eradication and for elaborating criteria of the health microbiome recovery.

### Microbiome analysis in lung and maxillary sinus samples

**Fig. 3:**
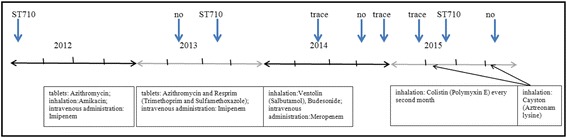
Time frames of therapy and Bcc control of patient 10. First row – Bcc control time (blue arrow): ST710 – some MLST targets detection, no – absence of PCR products in MLST analysis, trace – trace Bcc quality, detected only by *recA* gene primers. Second row – years of control. Third row – therapy. Black arrow points the month of the therapy

Patient 10 (P10) was under complex control since 2012. The main infecting bacterium was *Mycobacterium abscessus*, and aggressive therapy was directed at it (Fig. 3). In the beginning of 2012, *B. cenocepacia* ST710 was revealed in the patient’s sputum. After one year of treatment, *Bcc* was not detected by MLST, but a small concentration of it was present one month later. In May 2014, we detected only a trace of *Bcc* with the help of *recA* gene amplification, which is the most sensitive among *Bcc* MLST targets. Since July 2014, growth of *M. abscessus* on special medium has not been detected. Therapy became less aggressive and shorter duration. The last six sputum samples (Fig. 3) have been checked by MLST, and by massively parallel sequencing of *16S rDNA* V1-V4 hypervariable regions to analyze the steps of microbiome recovery.

**Fig. 4:**
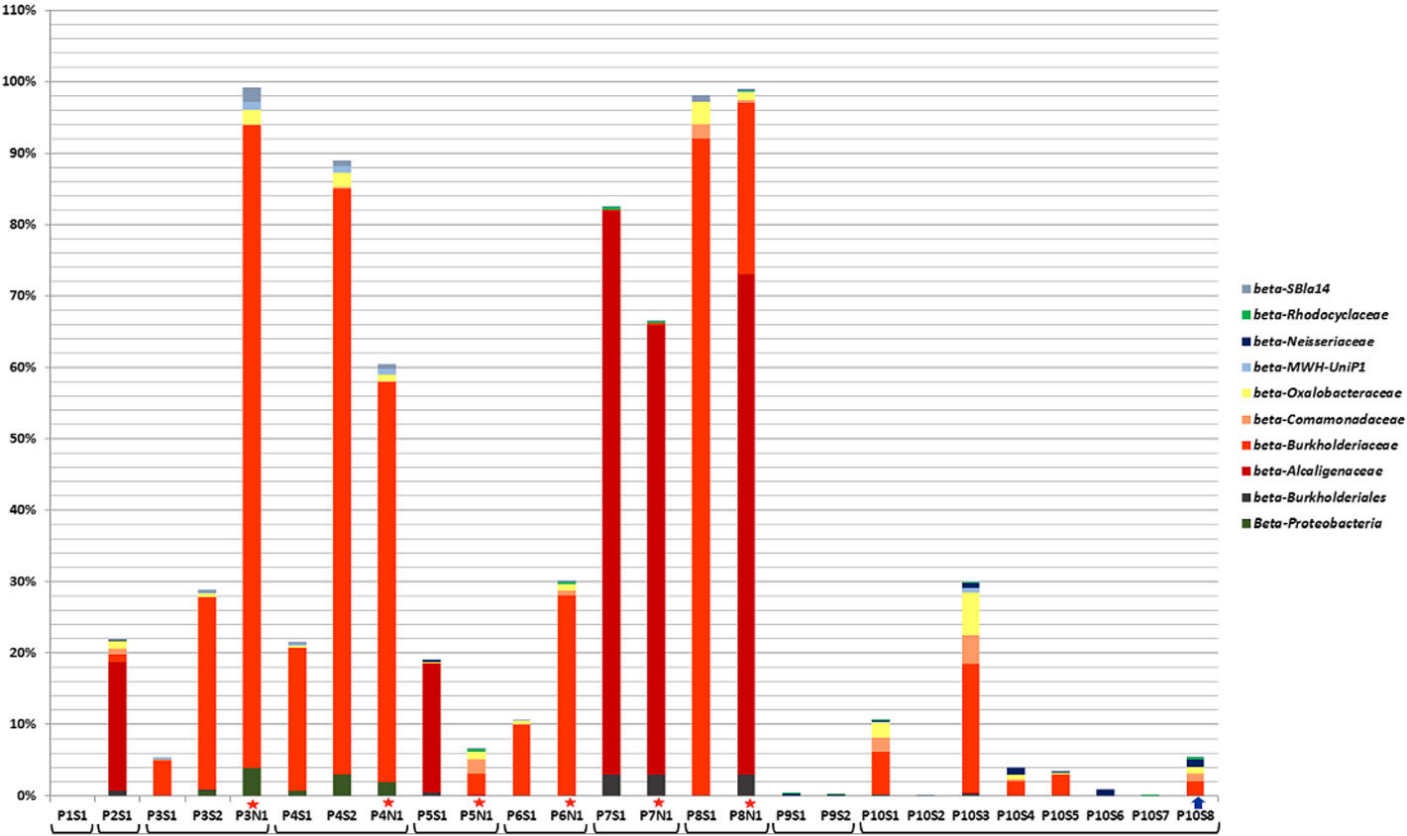
Betaproteobacteria families in patients’ microbiome based on *16S rDNA* sequencing data. S – sputum, N – maxillary sinus lavage. The brackets combined the samples from each patient. The stars indicate the samples from the sinuses. The arrow indicates the sample obtained during the patient 10 treatment with Ivacaftor

As depicted on Fig. 4, the proportion of Burkholderiacea in the bacterial community of sputum samples has changed from 0.0 to 18.0%. *16S rDNA* is a six-copies gene in the *Burkholderia* genome and is a more sensitive target than are one-copy genes of the MLST scheme. However, the results of MLST correlate with those of massively parallel sequencing.

**Fig. 5:**
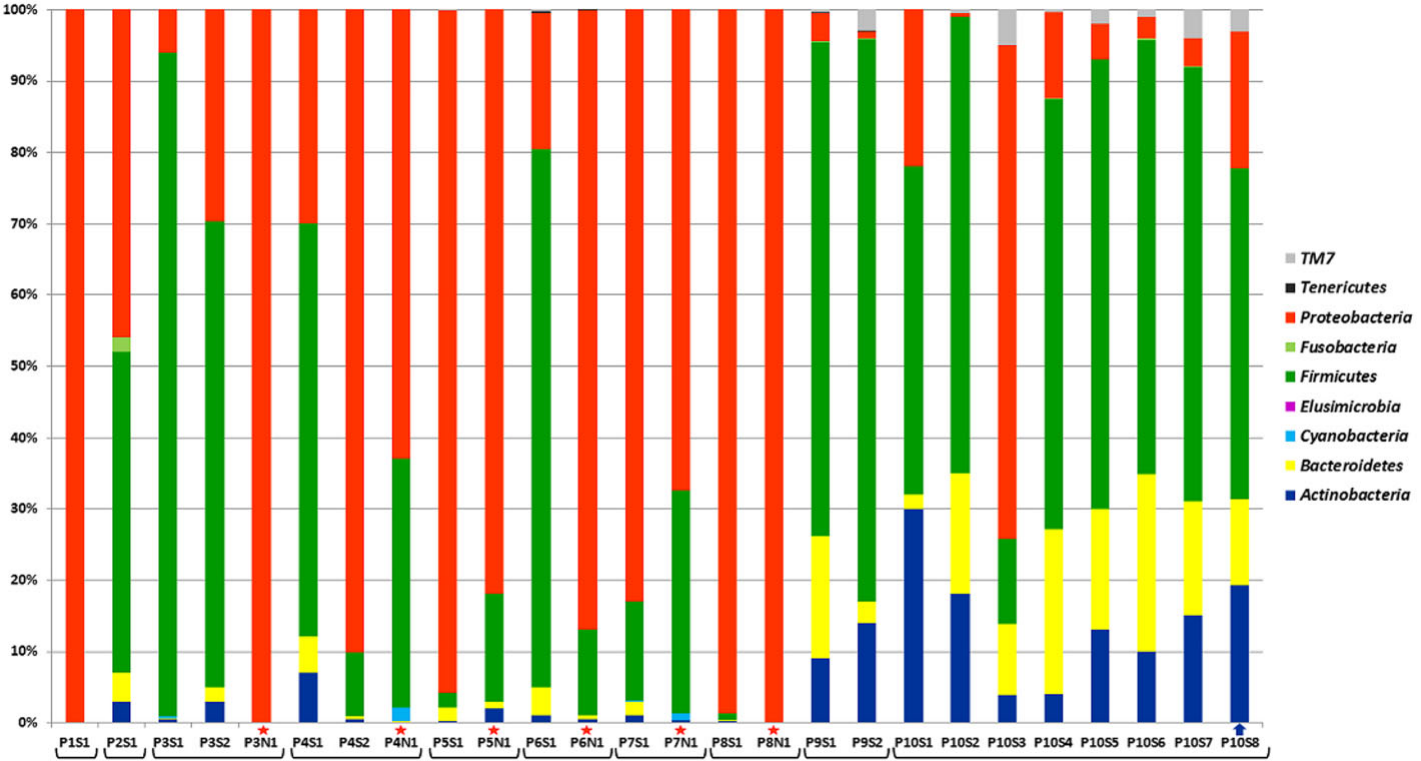
Microbial phyla in patients’ microbiome based on *16S rDNA* sequencing data. S – sputum, N – maxillary sinus lavage. The brackets combined the samples from each patient. The stars indicate the samples from the sinuses. The arrow indicates the sample obtained during the patient 10 treatment with Ivacaftor

**Fig. 6:**
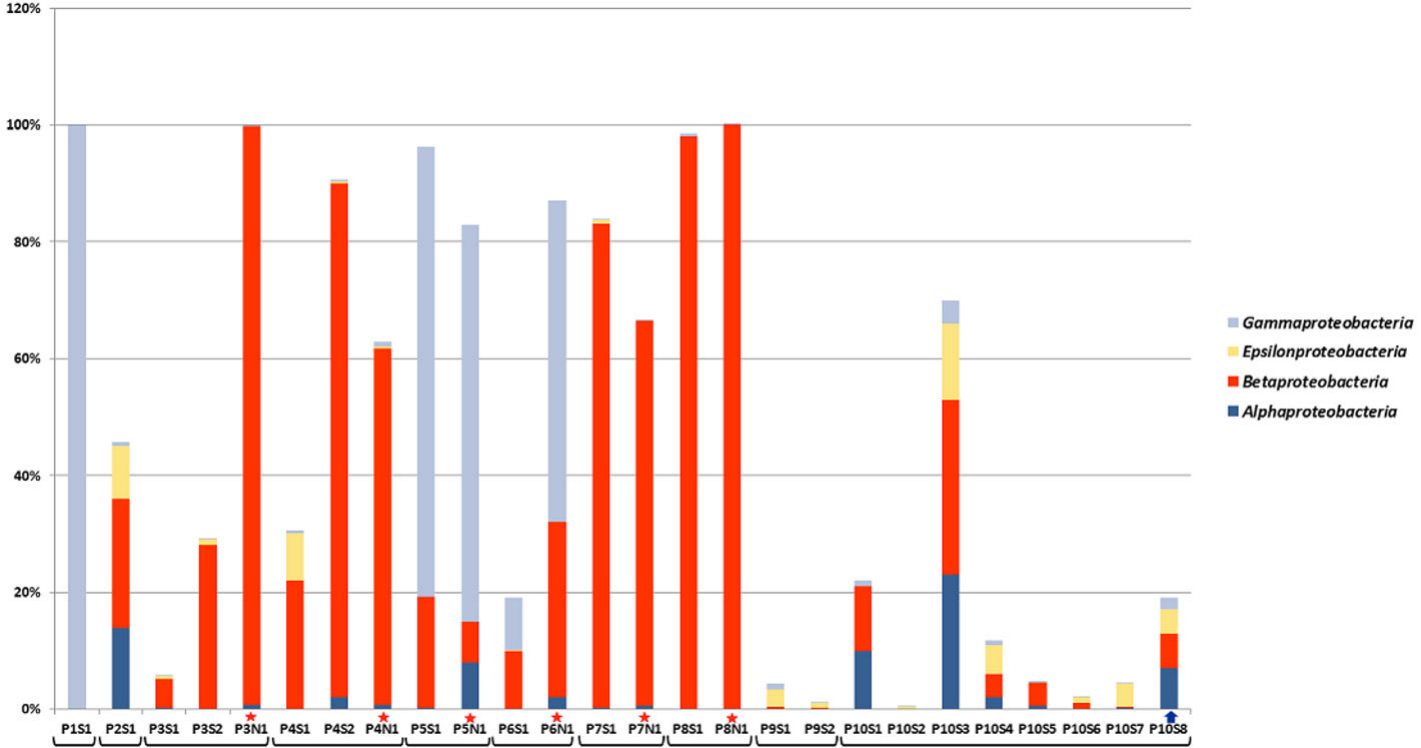
Proteobacteria classes in patients’ microbiome based on *16S rDNA* sequencing data. S – sputum, N – maxillary sinus lavage. The brackets combined the samples from each patient. The stars indicate the samples from the sinuses. The arrow indicates the sample obtained during the patient 10 treatment with Ivacaftor

Only trace amounts of *Mycobacterium* were present in samples P10S1 and P10S3 (0.149 and 0.257%), a finding which suggests that *M. abscessus* had been eradicated. According to Fig. 5, a “healthier” microbiome, corresponding to growth of Bacteroidetes, Firmicutes and Actinobacteria (but not Mycobacteria), has been established. Analysis of Proteobacteria phylum is illustrated in Fig. 6: the proportion of Epsilonproteobacteria in P10S3, P10S4, and P10S6 samples increased, and Neisseriales became the remarkable part of Betaproteobacteria class in the same samples (Fig. 4).

Thus, a central feature of a “healthier” microbiome in CF patients is that of increasing bacterial diversity, which is evident first at the phyla level.

Two last samples of P10 were associated with a new therapeutic approach in CF: treatment with Ivacaftor. Ivacaftor is a potentiator of the cystic fibrosis transmembrane conductance regulator (CFTR) and thus improves the transport of chloride through the ion channel. It was possible to use this treatment because P10 patient had N1303 K/G461E mutations in the *cftr* gene. The P10S7 sample was obtained days before start of therapy, and P10S8 was obtained after 4.5 months of therapy. As illustrated in Figs. 4, 5 and 6, the P10S7 sample had the “healthiest” microbiome. The P10S8 microbiome had representatives of various Proteobacteria classes and some families of Betaproteobacteria; in addition, trace amounts of Mycobacteria (0.158%) were present and *B. cenocepacia* ST710 was absent. These data are consistent with the view that Ivacaftor had improved the patient’s lung drainage. It must be noted the quick positive influence of Ivacoftor on the lung function of P10 patient (FEV1 increased from 50 to 67% during first 3 days of treatment, and slight increasing to 68% was observed during next 4.5 month, Additional file 1: Table S4). We consider this result as effect of the previous recovery of the microbiome diversity.

We compared P10 data with the results of chronically infected patients’ analyses. P1 was infected by *P. aeruginosa*; P2 by *P. aeruginosa* and *Achromobacter spp*.; P3 by *B. multivorans* ST835; P4 by *B. multivorans* ST712 and *P. aeruginosa*; P5 by *P. aeruginosa* and *A. ruhlandii* ST261; P6 by *B. cenocepacia* ST709 and *P. aeruginosa*; P7 by *A. ruhlandii* ST261; P8 by *B. cenocepacia* ST709 and *P. aeruginosa*. P9 was not chronically infected but had *A. ruhlandii* ST261 detected once.

In the group of 9 patients P9 had the “healthiest” microbiome and no bacteria in maxillary sinus lavage. The next stable patient was P3. Massively parallel sequencing (Fig. 5) suggested the prevalence of Firmicutes in P3S1 and P3S2, but demonstrated a small “healthy part” of microbiome, id est., Bacteroidetes and Actinobacteria. However, maxillary sinus lavage analysis of this patient (P3N1) demonstrated abundant Burkholderia, and MLST suggested *B. cenocepacia* ST835. The last 5 patients (P4N1-P8N1) had more than 50% of Proteobacteria in maxillary sinus lavage, and the main pathogen had the same ST as the pathogen from sputum; these data suggested the maxillary sinus can be an additive source of infection in CF patients. Comparing representatives of Firmicutes in sputum and sinus lavage samples we noted the prevalence of Streptococcaceae (up to 82%) in sputum microbiome, and presence less than 7% of Staphylococcaceae. However the proportion of Staphylococcaceae and in particular *Staphylococcus aureus* was increased only in maxillary sinus samples (up to 29%) of two patients.

Among all the samples we registered, Proteobacteria and Firmicutes were the most abundant phyla, reaching 100% and 93% of the microbiome, respectively. As a rule lower amounts of Proteobacteria were associated with higher proportions of Firmicutes (correlation coefficient was − 0.95), which probably reflects the competitive relationship between these phyla. In general, the presence of proteobacteria was associated with the suppression of other taxonomic groups of microorganisms and a decrease in microbial diversity. This, a moderate negative correlation was observed between the phyla Proteobacteria and Actinobacteria (− 0.65) and the phyla Proteobacteria and Bacteroidetes (− 0.67). This observation suggests that Proteobacteria is the most dangerous taxon in CF patients’ respiratory tracts.

## Discussion

*Burkholderia cepacia* syndrome is famous, and *B. cenocepacia* clone of ST234 has intercontinental distribution; the Russian epidemic strain BcenST709 is a member of this clone. Another famous representative of this clone is *B. cenocepacia* J2315 (ST28). This strain was isolated from CF patient, infected by the most notorious epidemic *B. cenocepacia* lineage ET-12. It has been revealed that ET-12 comprised a group of strains that have been causing devastating infections in Canadian, UK and European CF populations [56]. There is less information about the distribution of *A. ruhlandii*. Of strains submitted to the *Achromobacter* MLST database, 17.6% belong to this species [17]. The most famous is Danish epidemic strain, DES, *A. ruhlandii* A83/DSM25711 [57]. We found only 4 incomplete genomes of *A. ruhlandii* in GenBank (Assembly GCA_001637085.1, GCA_001637095.1, GCA_001637105.1, GCA_001637115.1); all of them were submitted by scientists from Brazil (Universidade do Estado do Rio de Janeiro), the country with the highest *Achromobacter* infection rate among CF patients: 21.0%, with 23.9% among children [58].

**Fig. 7:**
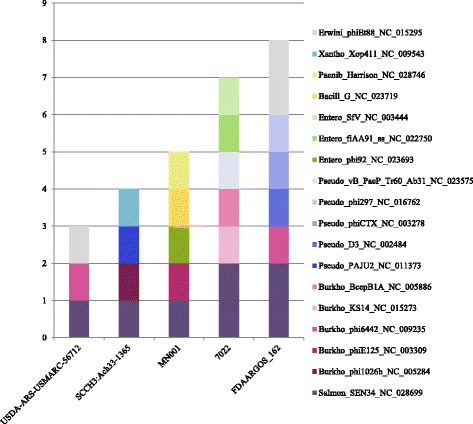
Prophage diversity in *A. ruchlandii* genomes

However, some complete *Achromobacter* genomes submitted as *A. xylosoxidans* or *A. denitrificans* were determined to be *A. ruhlandii* on the base of *Achromobacter* PubMLST server analysis [17]. This result is evidence of the necessity of strain verification based on new species criteria. Four strains form the NCBI Genome database were the object of our analysis: Three strains were isolated from CF patients (*A. xylosoxidans* MN001, CP012046.1, USA; *A. xylosoxidans* FDAARGOS_162, CP014065.1, USA; and *A. ruhlandii* strain 7022, NZ_LVKN01000001:NZ_LVKN01000089, Brazil), and one strain (*A. denitrificans* USDA-ARS-USMARC-56712, CP013923.1, USA) was from a domestic cow with respiratory disease. All strains were identified as *A. ruhlandii* on the base of MLST analyses. Strains *A. xylosoxidans* MN001 and *A. ruhlandii* 7022 had the same ST36 as that of the Russian epidemic strain AruhST36. *A. xylosoxidans* FDAARGOS_162 differed in 4 positions of allelic profile, and *A. denitrificans* USDA-ARS-USMARC-56712 had a new ST with new alleles of two loci. We compared prophage profiles of ipso facto *A. ruhlandii* strains; one common prophage typical for all these strains was revealed (Fig. 7). The fewest prophages were in a strain isolated from domestic cow. All clinical strains had specific prophages as individual markers. Thus, we conclude there is intercontinental spread of *A. ruhlandii* also. It should be noted that strains from Russia, USA and Brazil, which had the same ST36, were isolated from CF patients. This fact suggested the danger of *A. ruhlandii* ST36.

Multidrug resistance of Russian epidemic strains is main problem of these stains eradication. Genomic data suggested the huge resistant potential of strains. We compared obtained data with resistance of well-known CF pathogens *B. cenocepacia* J2315 and *A. xylosoxidans* NH44784–1996. Holden et al. [59] revealed at least four β-lactamases encoded in the J2315 genome, including: two class A, one class C, and one class D. Genes coding these β-lactamases were located on the chromosomes 2 and 3. However in BcenST709 there was more number of β-lactamase genes, and these genes belonged to 4 different classes. Moreover 5 from 13 β-lactamase genes were located on the chromosome 1.

The number of β-lactamase genes in *A. xylosoxidans* NH44784–1996 genome was 12. It was 2 greater than in AruhST36 [60]. One gene - *oxa-114*, coding β-lactamase of class D, had species specific allele. In *A. ruhlandii* this gene had allele 258.

A characteristic of AruhST36 T3SS is additional evidence of its danger, id est., as mentioned above, the presence of T3SS family Ysc in AruhST36 is indicative of an extracellular pathogen [53]. NCBI draft Genome data indicate that the T3SS family Ysc is present in two more *Achromobacter spp*. strains: one strain (*Achromobacter sp*. 2789STDY5608607, NZ_CYSX01000006.1) was isolated from CF sputum, the other (*A. xylosoxidans* KCJK1737) from *Bos taurus*.

Moreover, this type of T3SS family is typical of another Betaproteobacteria - *Bordetella* species. In *B. pertussis, B. parapertussis*, and *B. bronchiseptica* a T3SS cluster was found and identified as locus *bsc* [61]. This cluster included 30 ORF encoding T3SS genes, BscN ATPase, type III secreted proteins, and putative chaperones. The T3SS allows *B. bronchiseptica* to avoid phagocytosis and reduce the proinflammatory response [62]. At least in *B. bronchiseptica*, the Bsc system favors long-term colonization of the respiratory tract epithelium and downregulation of the immune response [62, 63].

BcenST709 T3SS belongs to another family, id est., Hrc-Hrp2. NCBI BLAST analysis of the T3SS gene cluster revealed other *B. cenocepacia* genomes with similarity in this area (Additional file 4: Table S3). The largest coverage (97–99%) and similarity (99%) of the T3SS cluster were found in strains isolated mainly from CF patients (indicated at the top of the table). The *B. cenocepacia* strains isolated from the environment (except one, CP013452.1) have less coverage (76%) and similarity to the T3SS cluster (90%). This information suggests that spread of pathogenic *B. cenocepacia* strains occurs among CF patients.

However, Russian physicians have demonstrated the ability to eradicate less dangerous *B. cenocepacia* strain. Microbiome analysis suggested that massively parallel sequencing of *16S rDNA* is a valid method for the appreciating therapeutic efficacy.

The case of patient 10 (P10) demonstrated that eradication of *B. cenocepacia* ST710 can result in recovery of microbiota diversity, approximating the microbiota of CF infants, as illustrated by Madan et al. [64]: genera of Firmicutes, Bacteriodetes and only Neisseria from Proteobacteria were present in the respiratory microbiota of patients from birth to 21 months [64]. Madan et al. analyzed oropharyngeal swabs, having believed that these traditional for infants samples [65] accurately reflected the microbial populations in the respiratory tract. In our investigation of the tracheal aspirate the most of the two phyla, Actinobacteria and Bacteroidetes, representatives are considered a “healthy part” of the respiratory tract, and can be as much as 20% the bacterial community of CF infants’ lungs [66].

Microbiome observations of patient P10 demonstrate positive dynamics, the first, due to complex antibiotic treatment: eradication of *M. abscessus* and *B. cenocepacia* ST710 and recovery of microbiota diversity. The second positive effect was that of treatment with Ivacaftor (P10S8), which likely improved lung drainage and additional emission of different Proteobacteria. Another healthier microbiome was observed in the respiratory tract of patient P9. It was marked by higher proportion of Firmicutes (69–79%), Actinobacteria (9%–14%), and Bacteroidetes (17%–3%), and a smaller proportion of Proteobacteria (4–1%). The microbiomes of P9 and P10 were characterized also by the presence of the poorly explored candidate phylum, TM7. Some data refer to a negative role of TM7, associated with human inflammatory mucosal diseases [67]. However, in this study we found only an association between TM7 and abundant Actinobacteria, which is an obligate epibiont of an *Actinomyces odontolyticus* [66].

An important finding of this study, which began as a search for a constant source of Proteobacteria in CF patients, was that the patients’ sinuses may harbor higher concentrations of dominant microorganism than do the lungs. Even the stable patient P3 had an abundance of *Burkholderia* in the maxillary sinus lavage. Thus, although adult CF patients with chronic infection may have periods of “healthier” microbiota, *Burkholderia* in the paranasal sinuses may be continual source of aggressive bacteria, entering the lungs.

The respiratory tract of CF patients is a dynamic ecosystem, and complete recovery of “healthy” microbiome is a long-term task. This task is complicated by various aspects of the CF patient’s physiology, as summarized in the review of Dickson et al. [68]. One component may be gastroesophageal dysfunction, which is common among CF patients and accelerates the migration of microbiota to the lungs. On the other hand, impaired mucociliary clearance inhibits the elimination of microbes. The lung destruction dramatically decreases the surface area of lungs and favors microbial growth; Burkholderiales are well adapted to the injured respiratory tract. Thus, effective treatment of these bacteria will require carefully matched therapy, perhaps with new drugs, taking into account WGS data and metabolic pathways of Burkholderiales.

## Conclusions

Complicated and dynamic bacterial communities in the airway of CF patients have been revealed recently. The bacterial communities change over time, in response to disease progression and response to antibiotic therapies. Our data demonstrate the positive effects of *Bcc* eradication and the importance of controlling the microbiome of CF patients’ sinuses. Eradication of registered Russian epidemic strains is difficult because of the complicated huge genomes encoding numerous resistance and virulence factors. Reverse vaccinology, based on WGS data and bioinformatics analyses are leading to approaches for Burkholderiales treatment and the development of new vaccines. Moreover, a multi-omics approaches could reveal the potential of the microbiome and some features of bacteria-bacteria and bacteria-lung interactions. Host functional changes could be monitored as well as the metabolites originating from both the host and the microbiome. Thus, changes in the microbiome and the host may be simultaneously followed to provide a more comprehensive picture of the dynamic changes that occur during disease. Surveying respiratory tract samples of adult and, especially, infant CF patients must be longitudinal; such surveys permit temporal shifts in bacterial diversity during airway infection and reveal the predictors of lung disease states (inflammation, exacerbation, stable).

## Additional files

Additional file 1: Table S4. Characteristics of the patients, included in the microbiome analysis. FEV1,% - percentage of predicted forced expiratory volume in 1 s; SD - substantial decline; S – stable. (XLSX 13 kb)

Additional file 2: Table S1. Genome features and virulence factors of A. ruhlandii ST36. (XLSX 59 kb)

Additional file 3: Table S2. Genome features and virulence factors of B. cenocepacia ST709. (XLSX 61 kb)

Additional file 4: Table S3 B. cenocepacia strains with similar T3SS gene cluster. (XLSX 12 kb)

## Abbreviations

AruhST36: Strain *A. ruhlandii* SCCH3:Ach33–1365 ST36
Bcc: *Burkholderia cepacia* complex
BcenST709: Strain *B. cenocepacia* GIMC4560:Bcn122 ST709
CF: Cystic fibrosis
CRISPR: Clustered regularly interspaced short palindromic repeats
Flp: Fimbrial low-molecular weight protein
IS: Insertion sequence
MLST: MultiLocus Sequence Typing
N1: Maxillary sinus lavage number in microbiome analysis
ORF: Open reading frames
P1: Patient’s number in microbiome analysis
PCR: Polymerase chain reaction
PenA: Penicillin-binding protein 2
PER: Pseudomonas extended resistance
RND: Resistance-Nodulation-Division
S1: Sputum sample number in microbiome analysis
ST: Sequence types
T3SS: Type III secretion system
WGS: Whole genome sequencing
